# Characterization of the Halcyon^TM^ multileaf collimator system

**DOI:** 10.1002/acm2.12568

**Published:** 2019-03-19

**Authors:** Tze Yee Lim, Irena Dragojević, David Hoffman, Everardo Flores‐Martinez, Gwe‐Ya Kim

**Affiliations:** ^1^ Department of Radiation Medicine and Applied Sciences University of California San Diego San Diego CA USA

**Keywords:** dual‐layer, Halcyon, MLC, multileaf collimators

## Abstract

**Purpose:**

To characterize the stacked and staggered dual‐layer multileaf collimator (MLC) on the Halcyon^TM^ system.

**Methods:**

The novel MLC assembly was reviewed and compared to the widely used Millennium^TM^ 120‐leaf MLC system. We investigated the MLC positioning stability over 70 days using Machine Performance Check (MPC) data. We evaluated the leaf transmission, penumbra, leaf end effect, and leaf edge effect. Leaf transmission through distal, proximal, and both MLC layers was measured with a Farmer chamber, by comparing an open and a closed field. Leaf penumbra was measured using film for three different MLC‐defined field sizes. The leaf end effect was measured with sweeping gap fields of varying gap sizes defined by the distal MLC. The leaf edge effect was evaluated using the Electronic Portal Imaging Device (EPID) for the different banks, gantry positions, and collimator angles. Point dose measurements for 10 test plans were compared to dose predictions of two dose calculation model versions.

**Results:**

From MPC data, the largest measured MLC positioning accuracy deviation was within 0.1 mm. The proximal MLC exhibited greater deviations compared to the distal MLC. The distal‐and‐proximal‐combination had reduced inter‐leaf and intra‐leaf transmission compared to delivery with distal‐only. The measured leaf transmission was 0.41% for distal‐only, 0.40% for proximal‐only, and negligible for distal‐and‐proximal‐combination. The leaf end penumbra was wider compared to the leaf edge penumbra. The leaf end effect was measured to be −0.2 mm. The leaf edge effect showed minimal bank, gantry position, and collimator angle dependence. However, a systematic deviation between measurements and treatment planning system handling of the leaf edge effect was observed. The discrepancy between the measured and predicted dose in the 10 test plans improved with the latest version of the dose calculation algorithm.

**Conclusion:**

The characteristics of the stacked and staggered dual‐layer MLC on the Halcyon^TM^ system were presented.

## 1 INTRODUCTION

Beam shaping plays a central role in increasing the accuracy, efficiency, and quality of radiation treatments. Multileaf collimators (MLCs) have been used in radiotherapy over three decades,^1^, ^2^, ^3^, ^4^, ^5^, ^6^, ^7^, ^8^, ^9^ initially as beam shapers, eliminating heavy shielding blocks, and later for intensity modulated radiotherapy (IMRT) and volumetrically modulated arc therapy (VMAT).^10^ Various MLC designs have been described over the years, each version aiming to further improve the outcome and quality of radiation therapy.^4^, ^8^, ^11^, ^12^, ^13^ For IMRT and VMAT treatments, the dose delivered to the target volume is sensitive to leaf positioning and leaf transmission. Characteristics of a well‐designed MLC therefore are: low leaf transmission, small tongue and groove effect, small penumbra, accurate leaf positioning, and faster speed.^5^, ^8^, ^14^, ^15^

In May 2017, Varian (Varian Medical Systems, Inc., Palo Alto, CA, USA) released Halcyon^TM^, a new external beam accelerator platform designed with the intent to enhance the workflow efficiency in radiotherapy. Halcyon^TM^ has a dual‐layer MLC system in contrast to other single‐layer Varian MLC systems (such as the Millennium^TM^ 120‐leaf MLC and High Definition 120‐leaf MLC). This design offers fast beam modulation and substantially reduces leakage between MLC leaves. Halcyon^TM^ has no beam shaping jaws, with the MLC being the only beam shaping component. Therefore, MLC positioning and optimization are essential to ensure accurate dose delivery.

The Halcyon^TM^ commissioning process is straightforward and streamlined to allow for a short period of time from installation to treatment. While the Halcyon^TM^ beam output has been described,^16^ the unique stacked and staggered dual‐layer MLC has not been independently characterized. Detailed characterization of the MLC system can provide a deeper understanding of the system's limitations, and thus inform the quality assurance protocols needed to ensure accurate radiation deliveries.

The purpose of this study was to characterize and assess performance of the stacked and staggered dual‐layer MLC system on the Halcyon^TM^ linear accelerator. We measured the MLC positioning accuracy and reproducibility over time. We also evaluated the leaf transmission, leaf penumbra, leaf end effect, and the leaf edge effect. To determine clinical impact, we examined ten plans used in end‐to‐end tests.

In this study, we comprehensively described the characteristics of the novel Halcyon^TM^ stacked‐and‐staggered dual‐layer MLC. Overall, we found the Halcyon^TM^ MLC system to be compatible with allowing for a clinic to have a strong focus on treating with intensity modulation, as a result of the MLC system's accurate leaf positioning, substantially low leaf transmission, and high leaf speed enabling the use of high dose rates. Nevertheless, the maximum MLC‐defined field of 28 cm^2^ × 28 cm^2^ may limit its use in certain disease sites requiring large field coverage.

## 2 MATERIALS AND METHODS

**Figure 1:**
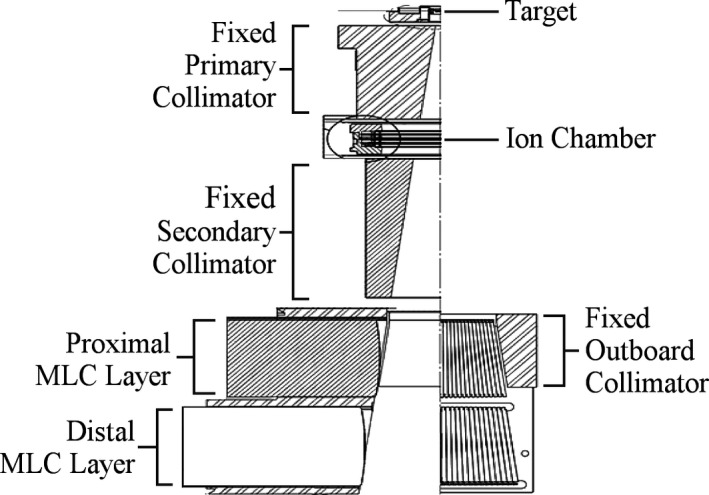
Schematic drawing of the Halcyon^TM^ head assembly.^18^ The primary and secondary collimators are fixed in place, and are not movable jaws. Shown are the positions of the proximal (upper) and distal (lower) MLC layers, with the bottom left displaying the leaf side view, and the bottom right illustrating the leaf end view.

The Halcyon^TM^ MLC system features a unique stacked‐and‐staggered dual‐layer design, consisting of a distal and a proximal layer.^17^, ^18^ The nomenclature for the MLC layers refers to their positions with reference to the source (Fig. 1). The primary and secondary collimators are fixed in place, and are not movable jaws. Also, note the absence of a flattening filter.

The distal, or lower layer, is comprised of two banks with 28 leaves each. The proximal, or upper layer, is comprised of two banks with 29 leaves each. The leaves are made from 95% tungsten (the remaining 5% is proprietary) and have a step edge. As shown in Fig. 1, the leaf ends form a truncated pie shape focused on the target, matching beam divergence in the direction perpendicular to the leaf travel (single‐focused). Each leaf measures 1.0 cm projected at isocenter with a leaf end curvature radius of 23.4 cm. The leaves are capable of 14 cm over‐travel (covering the entire field) and the leaf positioning tolerance is 1 mm. Table 1 details the comparison between this new Halcyon^TM^ MLC system^17^, ^18^ to the commonly available Millennium^TM^ 120‐leaf MLC system^19^, ^20^ by the same vendor.

**Table 1:** Comparison of the Varian Halcyon^TM^ MLC system^17^, ^18^ to the widely used Varian Millennium^TM^ 120‐leaf MLC system.^19^, ^20^

Characteristics	Halcyon^TM^ MLC	Millennium^TM^ 120‐leaf MLC
MLC Configuration		
Layers	Dual‐layer	Single‐layer
Beam shaping technique	Proximal and distal MLCs with 0.5 cm offset (no jaws)	MLC and jaws
Number of leaves	114 (29/bank on proximal, 28/bank on distal)	120 (60/bank)
Maximum field size	28 cm × 28 cm	40 cm × 40 cm
Direction of motion	Transverse	Transverse
Physical properties		
Leaf end shape	Rounded	Rounded
Leaf end radius	23.4 cm	8.0 cm
Leaf height	7.7 cm	6.5 cm
Leaf width (at isocenter)	1 cm	Pairs 1 & 40: 1.4 cm Pairs 2–10 and 51–59: 1 cm All others: 0.5 cm
Nominal 6MV‐FFF transmission	Single‐layer: 0.47% Dual‐layer: 0.01%	1.36%
MLC motion		
Leaf end position accuracy	1 mm	1 mm
Leaf velocity	5.0 cm/s	2.5 cm/s
Leaf acceleration	200 cm/s/s	50 cm/s/s
Position detection mechanism	Primary: Motor encoder Secondary: Soft pots	Primary: Motor encoder Secondary: Soft pots
Overtravel across central axis	14 cm	15 cm

### 2.A Positioning accuracy and reproducibility

Every day, as a requirement before the clinical use of Halcyon^TM^, a fixed series of tests under Machine Performance Check (MPC) is strictly enforced to establish the machine's proper function.^21^ A software interlock prevents user from running beam in clinical mode before MPC is completed and passed within vendor's preset tolerances (Table 2).

**Table 2:** Machine Performance Check (MPC) flags items for user review by comparing values acquired daily to the vendor‐specified tolerances from baseline

Categories	Items	Vendor‐specified tolerances
Isocenter	Size	±0.90 mm
	MV imager projection offset	±0.50 mm
Beam	Output change	±4.00%
	Uniformity change	±2.00%
Collimation	Rotation offset	±0.50°
Gantry	Absolute	±0.50°
	Relative	±0.50°
Couch	Lateral	±0.50 mm
	Longitudinal	±0.50 mm
	Vertical	±0.50 mm
	Virtual‐to‐isocenter lateral	±2.00 mm
	Virtual‐to‐isocenter longitudinal	±2.00 mm
	Virtual‐to‐isocenter vertical	±2.00 mm

Machine performance check includes a series of test to verify beam constancy and geometrical performance of gantry, collimator, and couch, using the vendor‐supplied phantom dedicated for Halcyon^TM^ MPC. One specific geometry test is the determination of MLC positioning accuracy and reproducibility.

Machine performance check evaluated MLC positioning accuracy by delivering a static comb pattern and acquiring the images using the 43 cm × 43 cm electronic portal imaging device (EPID) at gantry 0°. The measured distance between the MLC leaf end and the pre‐defined center of the MLC was compared to the intended distance. Furthermore, MPC evaluated MLC positioning reproducibility using a backlash pattern, where the MLC leaves shifted away from and then back to preset positions, acquired by the EPID. From 142 MPC reports (over 70 different days, as some days MPC was repeated), we recorded both the accuracy and reproducibility values for MLC positioning. An in‐house script was used to automatically detect and export MPC data each time MPC was performed. We analyzed the MLC positioning accuracy and reproducibility by grouping the leaves according to their respective layers and banks.

### 2.B Transmission

Average leaf transmission was measured for each leaf bank using a Farmer‐type ionization chamber (PTW 30013; PTW‐Freiburg, Germany). The chamber was positioned in solid water along the beam central axis with 100 cm source‐to‐surface distance (SSD) at two depths — d_max_ and 10 cm. Positioning of the Farmer chamber was verified by orthogonal megavoltage imaging. Measurements at d_max_ provided maximum dose collection while depth of 10 cm provided a more stable point of measurement. The fields were fully blocked using leaves entirely from each MLC bank — distal A bank, distal B bank, proximal A bank, and proximal B bank. When measuring transmission through each independent MLC bank, the opposing bank and both banks on the other layer were fully retracted. We also acquired chamber measurements under fields that were fully blocked using leaves from both the distal and proximal A banks, as well as leaves from both the distal and proximal B banks. To calculate transmission, the measurements acquired under each of these closed‐leaves deliveries were compared against an open field delivery.

To assess the inter‐leaf and intra‐leaf transmission, two picket fence deliveries were acquired using the EPID. The first picket fence delivery was defined with both MLC layers, with the proximal leaves always trailing the distal leaves during clinical deployment of the MLC system. The second picket fence delivery was defined with the distal leaves only (proximal leaves retracted) to independently evaluate the transmission for one layer. These two picket fence deliveries were repeated on ten separate days for stability evaluation. We then plotted the X and Y central axis profiles (averaged over the 10 days’ measurements) to compare the transmission between the picket fence delivery using both MLC layers versus a single MLC layer only.

### 2.C Leaf penumbra

The penumbras for three different field sizes (2 cm × 2 cm, 5 cm × 5 cm, and 10 cm × 10 cm) as shaped by the MLCs were measured on Halcyon^TM^ using the single available energy — 6 MV flattening‐filter‐free (FFF) beam. Gafchromic EBT‐XD self‐developing films (Ashland Advanced Materials; Bridgewater, NJ, USA) were positioned at a depth of 10 cm in solid water and source‐to‐axis distance of 100 cm. Since the beam on Halcyon is unflattened, penumbras were calculated according to method described by Ponisch et al.^22^ The profiles were normalized such that the inflection point, calculated as the maximum of the first derivative, corresponded to the 50% and then the 80–20% falloff distance was measured. We then compared the measured penumbra to the vendor‐reported penumbra (measured with diodes).^18^

### 2.D Leaf end effect

We used a Farmer chamber to determine the effect of the rounded leaf ends. The Farmer chamber was centered along the beam axis and verified using onboard MV imaging. We collected the readings from sweeping gap fields of various gap sizes (2, 4, 6, 10, 14, 16, and 20 mm) defined by the distal MLCs. Per vendor‐provided guidelines,^23^ after correcting for average leaf transmission's contribution to the gap readings, we extrapolated the various gaps corrected measurements down to 0 by fitting a linear function. The Y‐intercept of this linear fit provided the leaf end effect. Leaf end effect measurements were made at 100 SSD in solid water at d_max_ and then repeated for depth of 10 cm.

### 2.E Leaf edge effect

**Figure 2:**
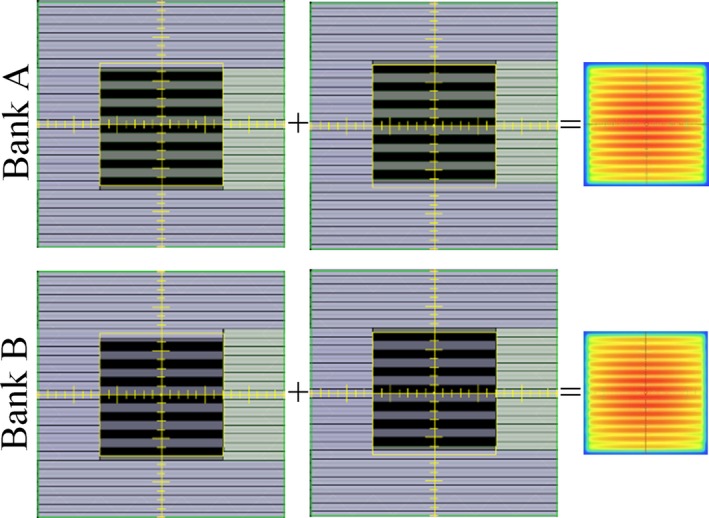
The leaf edge effect investigations used an MLC pattern of first closing the odd‐numbered distal MLCs, and then closing the even‐numbered distal MLCs. Shown in the figure are the deliveries of this pattern as defined by the two separate banks.

We investigated the leaf edge effects with the EPID using an MLC pattern that first extends the odd‐numbered distal MLCs and then extends the even‐numbered distal MLCs (Fig. 2). The EPID on Halcyon^TM^ is a Digital Megavoltage Imaging panel permanently mounted facing the source at a source‐to‐imager distance of 154 cm.^17^ For data acquisition, we used the “Portal Dosimetry” mode whereby the panel readout across the entire field was accumulated. The EPID was calibrated after TG‐51 reference dosimetry (cross‐check with optically stimulated luminescent detectors reported the ratio between absorbed dose determined by the Imaging and Radiation Oncology Core and our measurement to be 1.00),^16^ and the EPID output measurements agreed with Farmer chamber measurements to within 0.45% in a long‐term stability study.^24^

To investigate the dependency on the 2 MLC banks, the aforementioned MLC pattern was delivered using leaves from Bank A only, and then leaves from Bank B only. On these two EPID images, the central profiles perpendicular to leaf travel were normalized to dose maximum and compared.

To determine whether the leaf edge effect would be affected by gravity, this MLC pattern was also delivered at four different gantry positions (0°, 90°, 180°, and 270°). The central profiles perpendicular to leaf travel of the images acquired at the different gantry positions were normalized and compared.

Similarly, this MLC pattern was delivered at five different collimator angles (0°, 15°, 30°, 45°, and 90°). The central profiles perpendicular to leaf travel of the images acquired at the different collimator angles were normalized and compared.

We also compared the portal dosimetry measurements to the treatment planning system (TPS) predictions. The TPS used was Varian Eclipse (Varian Medical Systems, Palo Alto, CA, USA; Anisotropic Analytical Algorithm (AAA) versions 15.1 and 15.6).

### 2.F Clinical impact

We compared point dose measurements to TPS dose predictions for 10 VMAT plans. Two plans were generated for each of the five treatment sites investigated (prostate, head‐and‐neck, brain, gynecology, and spine). As part of the commissioning process, a point dose was measured with a PinPoint ionization chamber (PTW31014, Freiburg, Germany) in an IMRT homogeneous phantom (Model 002H5, Computerized Imaging Reference Systems, Norfolk, VA, USA) and compared to the TPS prediction (AAA 15.1). Upon the release of the next version — AAA 15.6, we recalculated the ten test plans to assess the effect of changes in MLC modeling on the performance of this new version compared to the previous version.

## 3 RESULTS AND DISCUSSION

### 3.A Positioning accuracy and reproducibility

**Figure 3:**
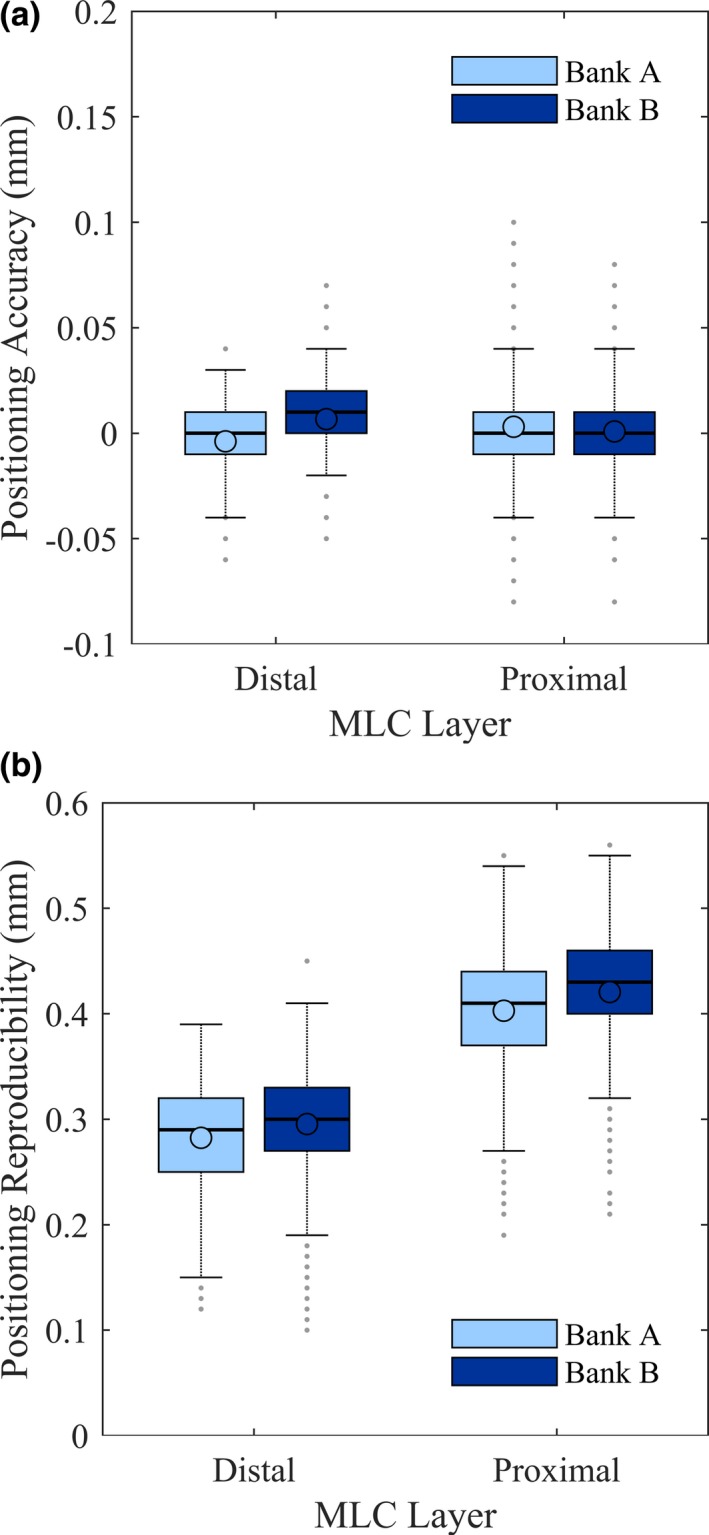
Range of MLC positioning (a) accuracy and (b) reproducibility over 142 Machine Performance Check runs. In each box, the circle indicated the mean, the central line indicated the median, while the top and bottom hinges indicated the 75th and 25th percentiles (values below which contain 75% and 25% of the measurements), the whiskers extended to the most extreme value within 1.5 times the interquartile range (difference between 75th and 25th percentile), and the dots extending past the whiskers indicated outliers.^27^

Figure 3 illustrates the range of MLC positioning accuracy and reproducibility of all leaves, grouped by MLC layer and bank. For MLC positional accuracy, the vendor‐specified tolerances of 0.6 mm for the distal leaves and 0.55 mm for the proximal leaves are much larger, given the observed maximum deviations of 0.07 mm for the distal MLCs and 0.1 mm for the proximal MLCs. Since MPC is mandatory for daily clinical use, Halcyon^TM^ users may study their own MPC data, as was done in this study. Li et al.^21^ reported on the appropriateness of MPC for Halcyon^TM^ daily QA in general and measured the MLC accuracy to be 0.05 ± 0.1 mm from intentional offsets of a single leaf. The users’ own readily available MPC data, the aforementioned study,^21^ and this work may be interpreted in the context of state and federal regulations to establish more stringent machine‐specific tolerances compared to the generous tolerances recommended by the vendor. Since the interface on Halcyon^TM^ disallow changing of tolerances, we use an external software (Image Owl; Total QA, Greenwich, NY, USA) to monitor the daily MPC trends and enforce the tolerances.

For MLC positional reproducibility, the vendor‐specified tolerance of 0.8 mm for the distal MLC and 0.9 mm for the proximal MLC is appropriate, given the observed maximum deviations of 0.45 mm for the distal MLC and 0.56 mm for the proximal MLC.

The pixel size on the EPID used was 0.34 mm physically and 0.22 mm when back‐projected to the isocenter plane.^17^ Therefore, users are recommended to be cognizant of the potential uncertainty when accuracy levels beyond the detector capability were to be reported by MPC.

### 3.B Transmission

Table 3 shows the leaf transmission for each layer independently, and for both layers. Specifically, note that no signal could be discernible from background noise when measuring the transmission through both layers.

**Table 3:** Leaf transmission for each MLC layer, and both MLC layers

	Leaf transmission (%)	
	d_max_	d = 10 cm
Distal‐only	0.41%	0.42%
Proximal‐only	0.40%	0.41%
Distal‐and‐proximal	Negligible	Negligible

**Figure 4:**
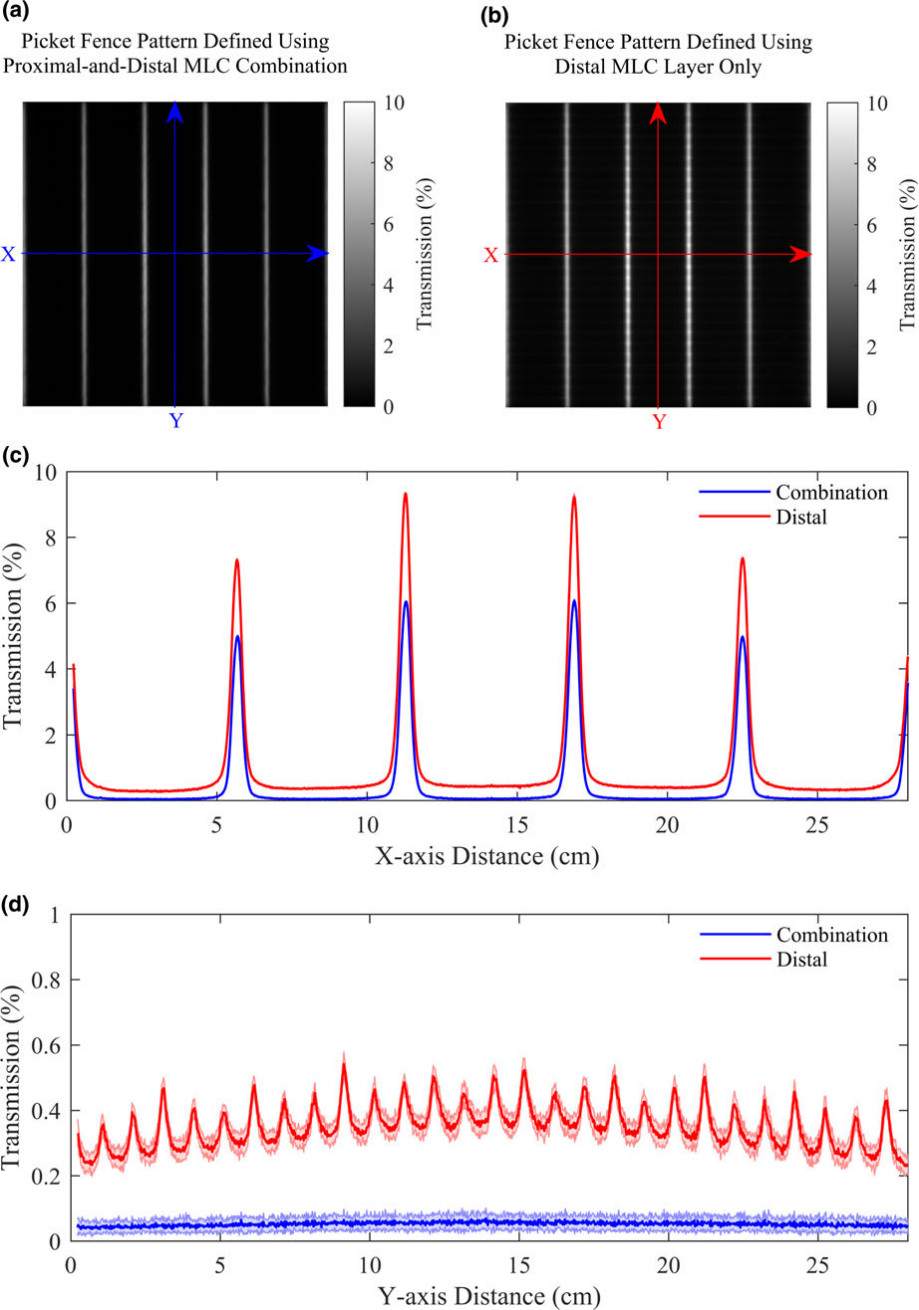
Picket fence patterns acquired using (a) both proximal and distal MLC layers, and (b) the distal MLC layer only (image acquired on the first day shown here). The central‐axis profiles were plotted across the (c) X‐axis and (d) Y‐axis, averaged over 10 days (standard deviation indicated by shaded areas). While the inter‐leaf leakage was clearly defined at 1 cm increments across the central axis of the dose distribution for the distal‐MLC‐only delivery, it was not discernible for the distal‐and‐proximal‐combination delivery.

For picket fence deliveries using the distal‐and‐proximal‐combination compared to distal‐only, the inter‐leaf and intra‐leaf transmissions were lower (Fig. 4). The inter‐leaf leakage peaks could be clearly identified at 1 cm increments for the distal‐only profile but were not discernible for the distal‐and‐proximal‐combination profile [Fig. 4(d)].

Since the transmission measurement at each depth was relative to the open field measurement at the same depth, the transmission values at both depths were similar. The measured transmission values of 0.40–0.42% for the Halcyon^TM^ MLC system closely matched the non‐user‐adjustable transmission value of 0.47% defined in the TPS. The measured transmission values for the Halcyon^TM^ dual‐layer MLC system met the AAPM TG‐50 and IEC requirements for average leaf transmission.^25^, ^26^

The Millennium^TM^ 120‐leaf MLC system has nominally < 2.5% average transmission and < 3% maximum transmission, and a transmission value of 1.36% has been reported.^19^ Therefore, the Halcyon^TM^ MLCs have significantly less transmission compared to standard Millennium^TM^ 120‐leaf MLCs. The single‐layer transmission of the Halcyon^TM^ MLC is similar to transmission of the Cobalt‐60‐based ViewRay MRIdian MLC^13^ and that of the Elekta Agility MLC when using a 6 MV beam.^4^

Dual‐layer delivery showed lower inter‐leaf and intra‐leaf transmission than single‐layer delivery. The lower inter‐leaf transmission for the distal‐and‐proximal‐combination delivery compared to the distal‐only delivery was due to the 0.5 cm staggering of the distal and proximal layers. Likewise, the lower intra‐leaf transmission for the distal‐and‐proximal‐combination delivery compared to the distal‐only delivery was a result of quantifying the transmission through two MLC layers versus only through one MLC layer.

### 3.C Leaf penumbra

**Figure 5:**
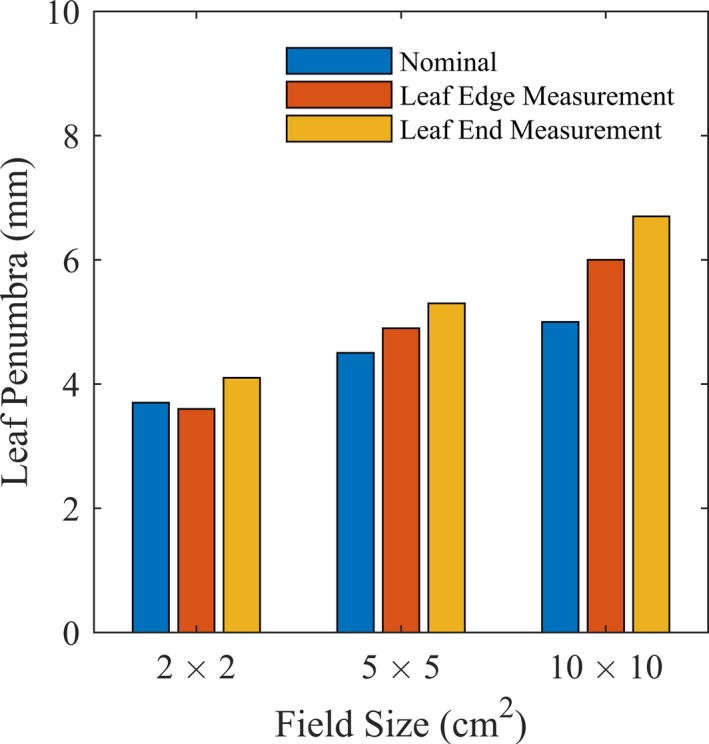
Measured penumbra at the leaf edges and leaf ends of the Halcyon^TM^ MLC compared to vendor‐reported values.^18^

Fig. 5 shows the leaf edge and leaf end penumbras measured on Halcyon^TM^ compared to vendor‐reported values.^18^ While the vendor‐reported measurements were performed at the same depth and distance from source, they were measured with diode (instead of film) and were the averages of the leaf edge and leaf end penumbra, potentially accounting for the difference between penumbra values reported by the vendor and in this study.

The leaf end penumbra was wider compared to the leaf edge penumbra, as the rounded Halcyon^TM^ MLC leaf ends resulted in decreased attenuation. Additionally, the penumbra width increased as a function of field size, most likely attributable to the diverging beam's varied attenuation across the rounded leaf ends as a function of distance from midline.

### 3.D Leaf end effect

**Figure 6:**
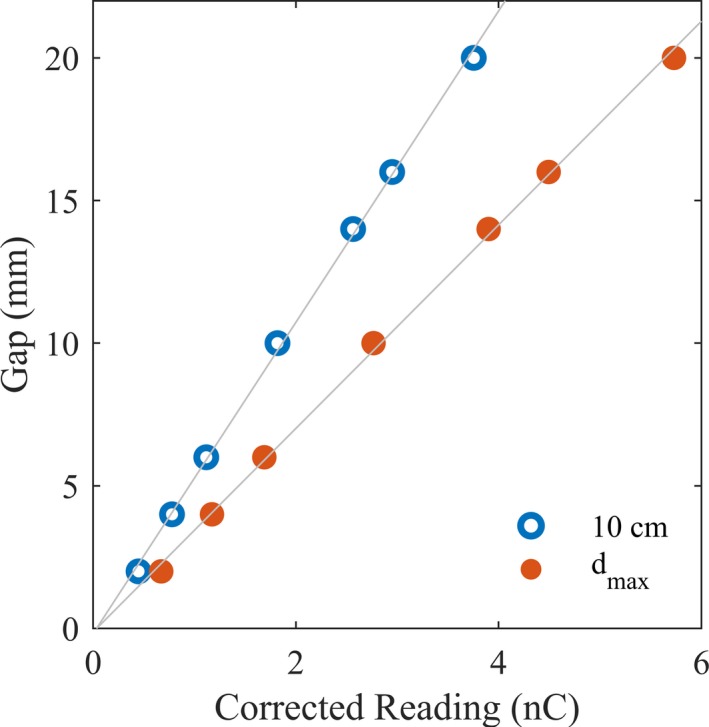
The leaf end effect, as informed by the Y‐intercept of the least square lines, was extrapolated from the transmission‐corrected chamber readings for the various gap sizes.

The measured leaf end effect was −0.19 mm from measurements at 10 cm depth, and −0.13 mm from measurements at d_max_ (Fig. 6). Suboptimal leaf end modeling reduces dose calculation accuracy and decreases the agreement between the predicted and measured dose. The measured leaf end effect values closely matched the non‐user‐adjustable value of 0.1 mm defined in the TPS for the MLCs. The difference in sign for the measured versus predicted leaf end effect suggested a difference in the overall dose direction when two leaf ends meet.

The leaf end effect presented was measured at the center of the field, but at off‐axis distances, we postulate that the leaf end effect would be affected due to decreased number of photons and increased photon travel distance. Investigation of the spatial variation in the leaf end effect would be an interesting future avenue of study.

### 3.E Leaf edge effect

**Figure 7:**
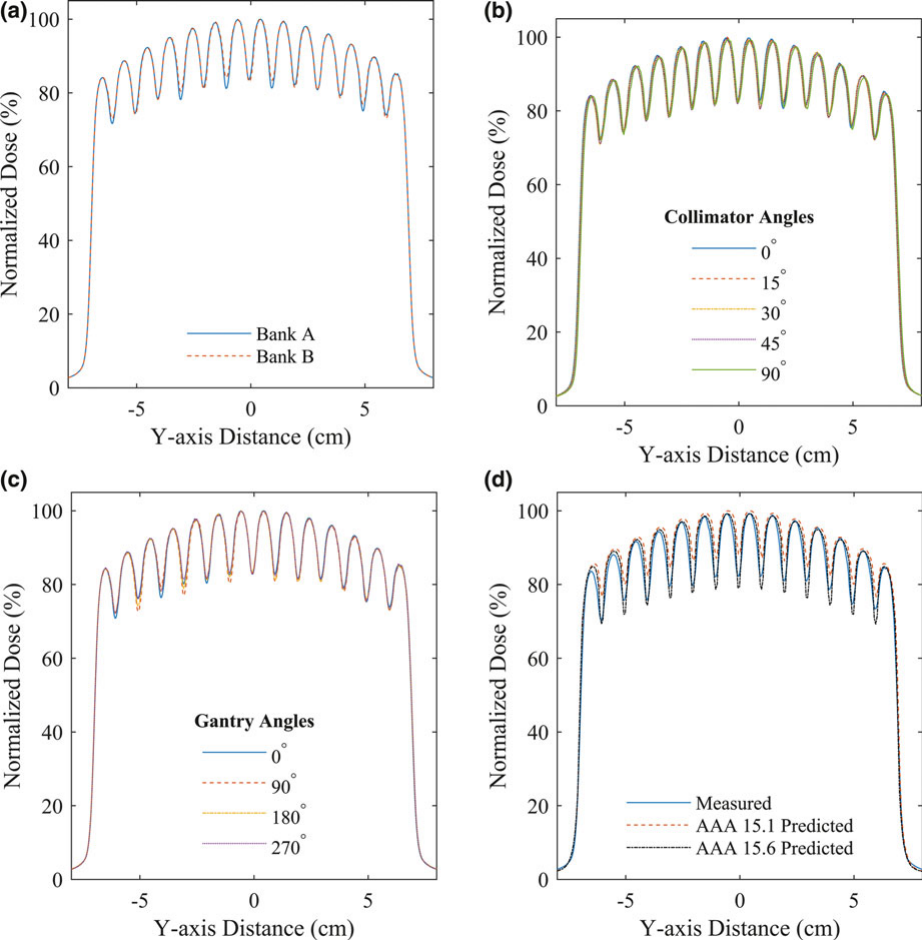
Profiles of the distal leaf edge measurements for different (a) banks, (b) collimator angles, (c) gantry positions, and (d) compared to the treatment planning system predictions.

The leaf edge profiles of Banks A and B were similar, with slightly larger dips between the leaf edges displayed by Bank A [Fig. 7(a)]. The leaf edge effect showed no clearly discernible gantry position and collimator angle dependence [Fig. 7(b) and 7(c)]. The similarity of the profiles for the different gantry positions and different collimator angles suggested minimal gravitational effects on the leaf edges’ dosimetry.

We observed a systematic discrepancy between EPID measurements and the TPS prediction using Eclipse AAA 15.1. The mean difference of underdosage defect arising solely from the leaf edge effect between the measurements and TPS predictions was −5.0% ± 1.1%. As can be seen in Fig. 7(d), the measured leaf edge effect was systematically deeper and shifted from the TPS prediction using Eclipse AAA 15.1, but improved with the next version — 15.6.

### 3.F Clinical impact

**Figure 8:**
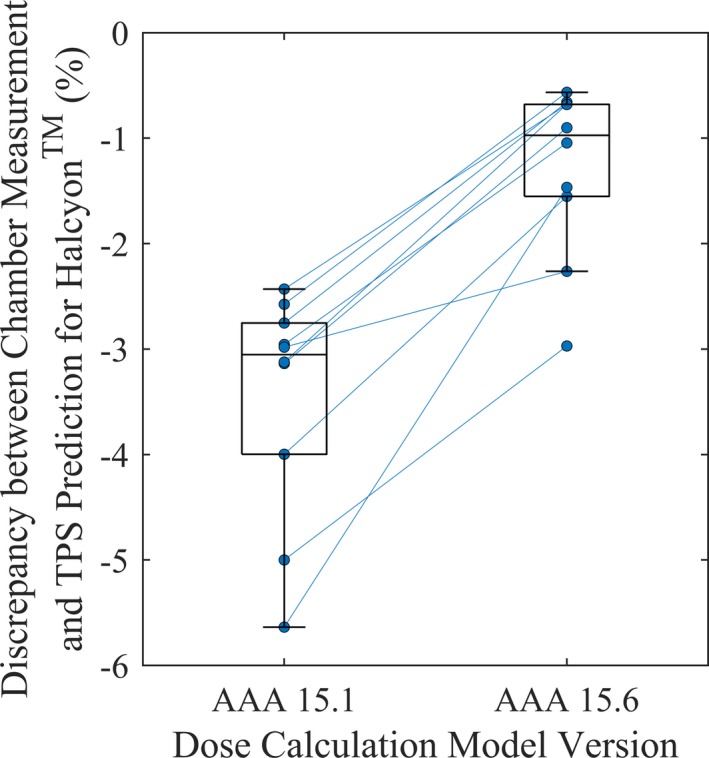
The discrepancy between measured dose and dose predicted the treatment planning system (TPS) for ten test plans delivered in phantom on the Halcyon^TM^ linear accelerator. Each circle represented the calculated discrepancies, with lines connecting the same plans to show the trend. Moving from an earlier to a later dose calculation algorithm — Anisotropic Analytical Algorithm (AAA) 15.1–15.6, the discrepancy between measurement and prediction was reduced from −3.46% ± 1.08% to −1.28% ± 0.80% (stated values are mean ± SD). In each box, the central line indicated the median, the top and bottom hinges indicated the 75th and 25th percentiles, and the whiskers extended to the most extreme value within 1.5× the interquartile range (difference between 75th and 25th percentile).^27^

Figure 8 shows the discrepancy between measured and predicted dose by the two TPS dose calculation model versions. These plans were delivered in phantom only and were not used for patient treatments. The magnitude of the dose discrepancy was dependent on the level of plan modulation. Minimally modulated plans displayed small discrepancies between measurements and predictions. Highly modulated plans usually have increased difference in the leaf travel distance between adjacent distal leaves, resulting in the trailing proximal leaves leaving some distal leaf edges exposed, potentially leading to the observed greater dose discrepancy. Overall, the measured leaf end effect showed a similar magnitude but opposite sign compared to the pre‐defined value in the TPS, coupled with the observed differences in measured and modeled leaf edge effect may have an impact on the delivery of highly modulated fields using Halcyon^TM^. Due to the dose discrepancy, the institutional practice for Halcyon^TM^ treatments were initially IMRT plans only, to allow for greater control of leaf travel during the planning stages. With the advent of AAA 15.6, we transitioned to VMAT planning. Addressing the discrepancy between measured results versus TPS predictions is the subject of ongoing investigation and communication with the vendor.

## 4 CONCLUSION

We have comprehensively evaluated the performance characteristics of the stacked and staggered MLC system on Halcyon^TM^. On the whole, the MLC system is advantageous for this era of intensity‐modulated treatments due to the high leaf positioning accuracy that is automatically monitored by the daily MPC, substantially low transmission even without jaws, and fast leaf speed enabling the use of high dose rates. Future work will investigate the TPS calculations and the correspondence to measured leaf end, leaf edge, and other dosimetric characteristics of the Halcyon^TM^ MLC system.

## CONFLICTS OF INTEREST

We have no relevant conflicts of interest to disclose.
